# Focus on your locus with a massively parallel reporter assay

**DOI:** 10.1186/s11689-022-09461-x

**Published:** 2022-09-09

**Authors:** Jessica C. McAfee, Jessica L. Bell, Oleh Krupa, Nana Matoba, Jason L. Stein, Hyejung Won

**Affiliations:** 1 Department of Genetics, University of North Carolina at Chapel Hill, Chapel Hill, NC 27599 USA; 2 UNC Neuroscience Center, University of North Carolina at Chapel Hill, Chapel Hill, NC 27599 USA

**Keywords:** MPRA, GWAS, Neurodevelopmental disorders, Noncoding genome, Gene regulation, Functional validation, Posttranscriptional regulation, Gene-environment interactions, WGS

## Abstract

A growing number of variants associated with risk for neurodevelopmental disorders have been identified by genome-wide association and whole genome sequencing studies. As common risk variants often fall within large haplotype blocks covering long stretches of the noncoding genome, the causal variants within an associated locus are often unknown. Similarly, the effect of rare noncoding risk variants identified by whole genome sequencing on molecular traits is seldom known without functional assays. A massively parallel reporter assay (MPRA) is an assay that can functionally validate thousands of regulatory elements simultaneously using high-throughput sequencing and barcode technology. MPRA has been adapted to various experimental designs that measure gene regulatory effects of genetic variants within cis- and trans-regulatory elements as well as posttranscriptional processes. This review discusses different MPRA designs that have been or could be used in the future to experimentally validate genetic variants associated with neurodevelopmental disorders. Though MPRA has limitations such as it does not model genomic context, this assay can help narrow down the underlying genetic causes of neurodevelopmental disorders by screening thousands of sequences in one experiment. We conclude by describing future directions of this technique such as applications of MPRA for gene-by-environment interactions and pharmacogenetics.

## Introduction

Genome-wide association studies (GWAS) of neurodevelopmental and psychiatric disorders have demonstrated that the majority of common variation associated with these disorders is found in noncoding regions of the genome [1–8]. Similarly, whole-genome sequencing studies (WGS) are poised to discover rare noncoding genetic variation associated with neurodevelopmental disorders [9–11]. Whereas the functional impact of genetic variation in protein coding regions can be inferred through knowledge of the codon code, the impact of genetic variation in the noncoding genome is much more difficult to understand as no such regulatory code is known. The noncoding genome contains cis-regulatory regulatory elements (CREs) such as enhancers, promoters, silencers, and insulators, which influence gene expression by serving as docking sites for DNA-binding proteins like transcription factors (TFs) [12, 13]. Variants within a regulatory element can alter TF binding and subsequently alter gene expression and cellular function [14, 15].

**Fig. 1:**
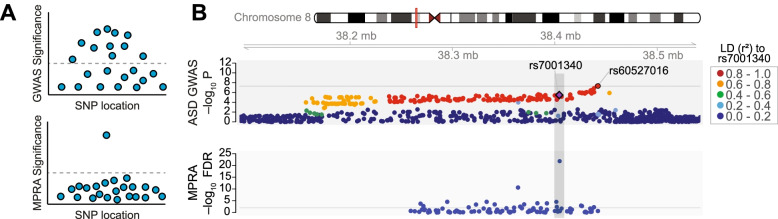
Use of MPRA to identify causal variants at a GWAS locus containing many SNPs in high LD. **A** The schematic cartoon plots show GWAS and MPRA SNPs and their corresponding significance at a single locus. LD structure confounds identification of the causal variant in the GWAS, but the MPRA tests regulatory effects of each SNP independently so it can identify a specific causal variant. **B**. Top, SNP association statistics at a genome-wide significant locus from an ASD GWAS [19]. The index SNP, rs60527016, reached genome-wide significance. SNPs are colored by binned LD (*r*^2^) relative to the MPRA-validated variant (rs7001340). The existence of SNPs that are in high LD with rs7001340 highlights the difficulties in defining which SNPs are functional or causal based on GWAS alone. Bottom, MPRA identified a causal variant within this locus (rs7001340) that shows strong allelic regulatory activity. Image adapted from [19]

In addition to the lack of regulatory code, GWAS alone cannot pinpoint variants that are causing a disease because of linkage disequilibrium (LD), the nonrandom inheritance of nearby alleles on the genome. A genome-wide significant (GWS) locus typically contains tens to hundreds of single-nucleotide polymorphisms (SNPs) that are associated with a trait or disease. Only a subset of these SNPs are thought to be causal. While it is commonly thought that the index SNP, the SNP most significantly associated with the trait at a locus, is causal, growing evidence portrays a more complex picture [16]. The lead SNP is not always the causal allele when functionally validated, and a given locus can contain multiple causal variants [16]. Identifying the causal variant(s) at a locus can greatly facilitate our understanding of disease mechanisms by narrowing down the genetic underpinnings of a disease (Fig. 1). Moreover, causal variant identification provides the intriguing possibility of developing therapeutics by reversing pathological transcriptional mechanisms or genetically modifying causal variants [17, 18].

Several experimental and computational designs have been used to predict the causal variant at a given locus. Fine-mapping tools computationally predict potential causal variants based on association statistics and LD patterns [20–23], but different algorithms can yield conflicting results, and prioritized variants still require experimental validation [24]. Allele-specific chromatin accessibility (ASCA) can be used to determine if inherent genetic variation in a population of individuals affects chromatin accessibility, a proxy for gene regulatory activity, in relevant cell types [25, 26]. Genetic variants within a noncoding regulatory element that affects the function of that element are highly likely to be causal gene regulatory variants. Allele-specific chromatin accessibility when colocalized with GWAS suggests that the genetic variants are also causally associated with the trait or disease. However, ASCA experiments require large sample sizes of genetically diverse donors with both genotype and chromatin accessibility data, and they cannot independently test the effect of multiple variants in high LD within a regulatory element [27, 28].

**Fig. 2:**
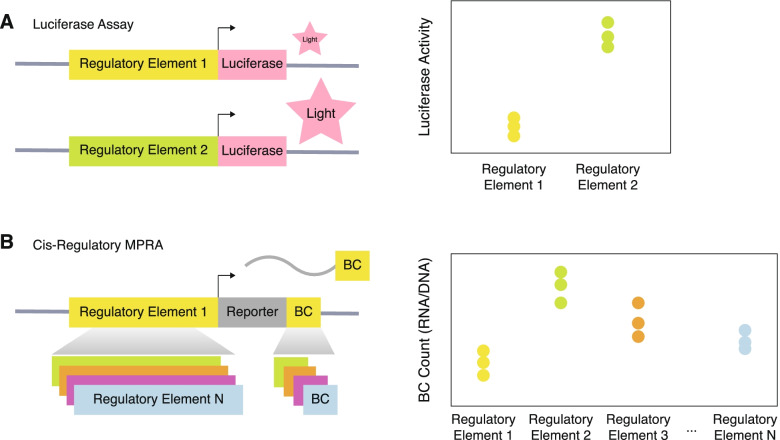
Luciferase assay vs cis-regulatory MPRA. **A** Luciferase assay measures light emitted by a reporter gene, luciferase, driven by a regulatory element. **B** In a canonical cis-regulatory MPRA, the regulatory element drives the RNA expression of the unique barcodes. Transcriptional activity is quantified as barcode transcription (via RNA-seq of the barcodes) normalized to initial input of barcodes (via DNA-seq of the barcodes). Thousands of cis-regulatory elements (CREs) can be tested in the same experiment

Functional validation assays fill the existing gap by experimentally demonstrating how genetic differences lead to phenotypic effects [29]. Gene regulatory activity of noncoding elements has historically been functionally validated via luciferase assays (Fig. 2A). A luciferase assay places a regulatory sequence of interest (sometimes containing a SNP) upstream of a luciferase reporter gene and quantifies the regulatory effect on expression via luminescence of luciferase [27]. However, luciferase assays lack the throughput to validate thousands of regulatory sequences at once because each regulatory element must be measured independently.

Massively parallel reporter assays (MPRA) have advanced the throughput of luciferase assays by enabling the simultaneous functional validation of regulatory activity of thousands of variants on a massive scale (Fig. 2B), often vastly narrowing down thousands of variants found by GWAS or quantitative trait loci (QTLs) in a single assay (Fig. 1). Rather than quantifying the luminescence of luciferase, MPRA measures the barcoded reporter gene expression via next-generation sequencing. Once the MPRA construct is introduced into cells of interest, the synthesized regulatory element drives the expression of its unique barcode, a random oligo sequence that uniquely tags the matching regulatory element. The initial input of the construct is quantified by the DNA counts of the barcodes, which is compared to the RNA counts to evaluate effects on expression (Fig. 2B).

MPRA has incredible potential for studying the noncoding genetic variants associated with neurodevelopmental disorders. Whereas the majority of efforts have been made to characterize common variants identified by GWAS, wide application of WGS would further expand the utility of MPRA in characterizing various classes of variants located in the noncoding genome.

In this review, we will discuss the broad application of MPRA to functionally validate variants within the various regulatory contexts that encompass transcriptional and posttranscriptional regulation. We will then add important considerations for conducting MPRA, including limitations of MPRA experiments. We conclude by providing future directions of MPRA.

### MPRA for studying cis-regulatory elements

#### Canonical MPRA

The canonical MPRA design includes a CRE, a generic promoter, a reporter gene, and a unique barcode assigned to each regulatory element (Fig. 2B). Generally, CRE libraries of interest are made with mass oligonucleotide synthesis. To interrogate variant effects on gene regulation, the CRE can be modified to harbor a variant within its sequence. Additionally, every possible single-nucleotide mutation can be added to the CRE, called saturation mutagenesis. The impact of the variant on regulatory activity is measured through barcodes matched to each unique variant. Because the barcode itself can have an influence on levels of expression, many barcodes are usually tested for each variant. Transcriptional activity is quantified as barcode transcription (via RNA-seq of the barcodes) normalized to initial input of barcodes (via DNA-seq of the barcodes). This allows systematic investigation of variant function within a noncoding region by comparing the gene regulatory activity between protective and risk alleles of a given variant. A growing body of research employs this strategy to identify functional regulatory variants within GWAS loci [30–34]. Key consideration in designing MPRA involves the use of proper controls [35]. For example, scrambled sequences of DNA in the relevant cell type can be used as negative controls to experimentally validate enhancers [35]. Likewise, a strong promoter or a known highly expressed sequence in the relevant cell type can be used as positive controls [35]. The canonical MPRA design has been adapted to fit the needs of differing types of CREs being tested [36–39].

#### Promoter

**Fig. 3:**
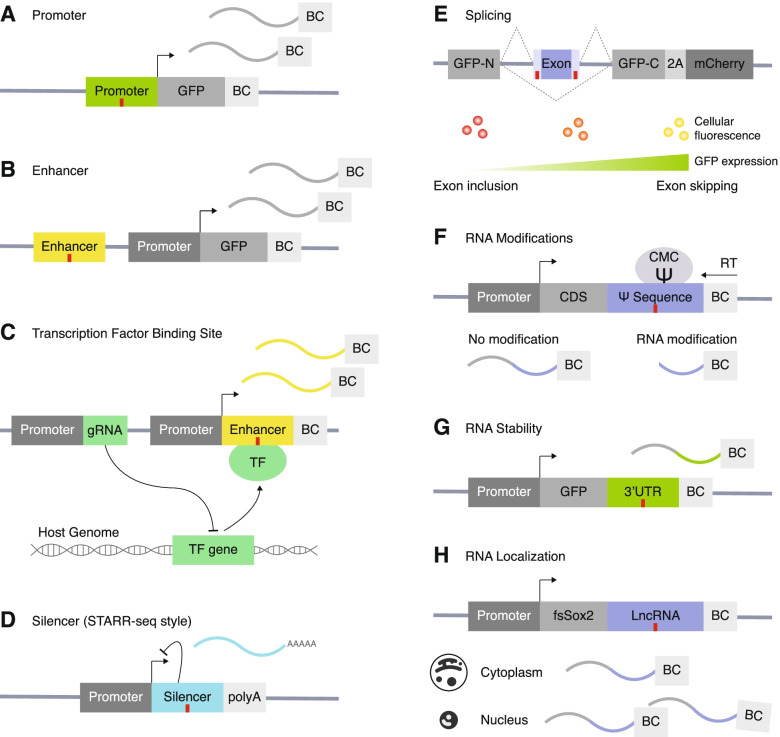
MPRA designs for studying gene regulation. MPRA modifies the design of canonical cis-regulatory MPRA (described in Fig. 2B) that contains a cis-regulatory element (CRE), a promoter, a reporter gene, and a unique barcode (BC). Elements of this construct can be replaced or rearranged to test different types of CREs. The red vertical line indicates where a variant can be located. **A** Promoter MPRA contains a promoter harboring a variant, a reporter gene (e.g., GFP), and a unique BC. Image adapted from [40]. **B** Enhancer MPRA contains a regulatory element harboring a variant, a (minimal) promoter, a reporter gene, and a unique BC. **C** Transcription factor binding MPRA (TransMPRA) can be broken down into two components: (1) a promoter with a guide RNA (gRNA) that targets a transcription factor (TF) of interest and (2) a promoter, a test enhancer sequence harboring a variant, and a unique BC. The gRNA brings catalytically dead Cas9 protein with an attached Krüppel-associated box (dCas9-KRAB) which silences the expression of the TF gene. If the silenced TF interacts with the test enhancer, the downstream barcode expression is decreased. Image adapted from preprint [41]. **D** Silencer MPRA (in a STARR-seq style) contains a (strong) promoter and a test silencer harboring a variant. The silencer sequence can prevent self-transcription by silencing the promoter. Image adapted from [42]. **E** Splicing MPRA has minigene constructs that are inserted between a split-GFP reporter (GFP-N terminus and GFP-C terminus) and a peptide 2A (P2A) upstream of an mCherry reporter. Variants can be located in the variable intron sections on either side of the exon or within the exon. Inclusion of the middle exon disrupts GFP fluorescence, and cells can be FACS sorted into bins based on GFP:mCherry ratios. The GFP with or without the exon are quantified for exon inclusion or skipping via DNA-seq of the plasmid in each sorted bin. Image adapted from [43]. **F** RNA modification MPRA contains a promoter, an arbitrary coding sequence (CDS), a putative pseudouridine (Ψ) sequence as 3′ untranslated region (UTR), and a unique barcode. Once the library is introduced, cells are treated with N-cyclohexyl-N′-β-(4-methylmorpholinium) ethylcarbodiimide (CMC) which binds to Ψ and prevents reverse transcription (RT). High-throughput sequencing of cDNA allows prediction of the exact base pair location of the Ψ RNA modification. Variants can be inserted anywhere in the CDS. Image adapted from [44]. **G** 3′ UTR MPRA consists of a promoter, a reporter gene, a 3′ UTR harboring a variant, and a BC. BC RNA counts reflect transcriptional stability modulated by 3′ UTRs. **H** RNA localization MPRA consists of a promoter, a mutated *Sox2* gene that localizes in the cytoplasm (*fsSox2*), a lncRNA harboring a variant, and a unique barcode. Barcode expression from subcellular fractions is used to interrogate subcellular localization of lncRNA. Image adapted from [45]

Mutations and variants in promoter regions can have a profound impact on gene expression. MPRA has been used to test the impact of variants in promoters on a massive scale. In comparison with the canonical MPRA design, promoter MPRA lacks a CRE and alters the DNA sequence of the promoter region. Patwardhan et al. utilized promoter MPRA with saturation mutagenesis to screen the activity of mutated promoter sequences with attached barcodes (Fig. 3A) [40]. Barcode counts quantified via short-read sequencing provided a scalable readout of promoter activity, which led to the identification of critical regions of a promoter that govern transcriptional efficiency [40].

In addition to introducing variation in the promoter sequences, a similar approach can be used to characterize promoter activity of any given sequence. Boer et al. developed a gigantic parallel reporter assay (GPRA) that measured the promoter activity of over 100 million randomly synthesized sequences [46]. The complexity of synthetic promoters surpasses the complexity of the human genome, allowing them to build a predictive model of how genetic sequence affects transcriptional regulation.

While many neurodevelopmental disorder-associated variants have been shown to be enriched in promoter regions [24, 47, 48], MPRA has yet to be adopted to systematically examine the regulatory function of these promoter variants. We expect MPRA will provide a useful avenue to elucidate the function of promoter variants associated with neurodevelopmental disorders.

#### Enhancers

Enhancers are CREs that TFs bind to and activate gene expression [49]. Disease-associated risk variants are enriched in enhancers [50]. Despite their important roles in gene regulation and disease associations, the sequence logic of enhancers is not well understood. Therefore, MPRA has been widely adapted to experimentally test the function of enhancers and variants within enhancers [19, 51]. While MPRA can take on many forms to examine enhancer functions [52], generally, a putative enhancer element is coupled with a weak promoter (e.g., minimal promoter) that is followed by a reporter gene and a unique barcode (Fig. 3B).

Myint et al. used enhancer MPRA to screen 1049 schizophrenia- and 30 Alzheimer’s disease-associated variants for differences in driving reporter gene expression [34]. They used two cell lines and identified 192 SNPs with significant differences in driving reporter gene expression [34]. Among the 192 variants, 148 showed allelic differences in K562 cells, 53 in SK-SY5Y cells, and only 9 showed allelic differences in both cell lines, demonstrating that genetic variants often exert their regulatory effects only within specific cell types [34]. As an additional example, Matoba et al. used MPRA to fine-map one novel ASD GWAS locus in HEK293T cells (Fig. 1) [19]. Of 98 variants tested, two were found to have significant differential allelic activity, with one variant (rs7001340) exhibiting strong effects. By integrating MPRA results with expression quantitative trait loci (eQTLs), they showed that an ASD-associated risk allele decreased the expression of *DDHD2* [19]. These examples highlight MPRA’s ability to map disease-associated variants within putative enhancer regions.

Transcription factors (TF) recognize and bind to specific sequences within an enhancer, called TF binding motifs, to regulate gene expression. Variants within motifs can disrupt TF binding or create new motifs, altering regulatory activity. Though enhancer MPRA can identify if a variant affects enhancer activity, it does not experimentally validate which TF contributes to the altered regulation. TF-DNA interactions can be measured using methods such as chromatin immunoprecipitation sequencing (ChIP-seq), or they can be inferred using CRISPR knockout screens that model the impact of TFs on gene regulatory programs [53–55]. A recently introduced technique (in preprint) called TransMPRA also addresses this question by combining MPRA with CRISPR interference and single-cell sequencing to measure the interaction between transacting factors and putative enhancers (Fig. 3C) [41]. In this system, a guide RNA (gRNA) for a known TF is packaged together with enhancer MPRA that are potentially directly targeted by the TF [41]. When introduced into cells expressing dCas9-KRAB proteins, TF expression is inhibited, and enhancer activity is reduced only if the element is a downstream target of the TF [41]. Accordingly, TransMPRA provides an incredibly important tool to delineate potential transcriptional regulators for noncoding variants associated with neurodevelopmental disorders.

#### Silencers

MPRA has been adapted to test silencer elements, which are noncoding functional elements that lead to decreased expression of their target gene (Fig. 3D) [42]. Silencer MPRA differs from enhancer MPRA in two aspects. First, enhancer MPRA uses a weak promoter (e.g., minimal promoter) to measure increases in gene expression elicited by the putative enhancer, while silencer MPRA uses a strong promoter (e.g., super core promoter, SCP1) that transcribes a high baseline level of the construct, so decreases in transcription can be detected. Second, silencer MPRA leverages the design of self-transcribing active regulatory region sequencing (STARR-seq), a sub-branch of MPRA (for more information about STARR-seq, please see the review [56]). STARR-seq places an uncharacterized CRE downstream of a strong core promoter followed by a polyA tail. This MPRA design does not require barcodes because the sequence of the transcribed putative silencer acts as the barcode [42]. While MPRA in the STARR-seq style has been widely adopted, it is important to consider that mRNA sequences could be affected by posttranscriptional effects such as mRNA degradation which would be indistinguishable from transcriptional effects [57].

Silencer MPRA has been applied to detect thousands of CREs acting as silencers, which were enriched for disease-associated SNPs [42], highlighting the need to decipher regulatory logic of transcriptional silencing in understanding disease etiology [42].

### MPRA for studying posttranscriptional regulation

#### Splicing

MPRA can be combined with methods that sequence populations of cells binned by fluorophore expression, called Sort-seq [58], to study posttranscriptional processes like alternative splicing. In splicing MPRA, a red fluorophore (mCherry) is constitutively expressed, and a three-exon, two-intron minigene construct is cloned into a plasmid in such a way that when the middle (tested) exon is skipped, a green fluorophore (GFP) is also expressed (Fig. 3E) [43]. Variants can be located in the variable intron sections on either side of the test exon or within the exon. Cells are sorted into bins using GFP:mCherry ratios by fluorescence-activated cell sorting (FACS), where a higher ratio indicates greater intron excision. Plasmid DNA is then sequenced within each bin to determine which variants affect splicing. In an experiment utilizing this assay, many of the variants that lead to differences in splicing were located outside of canonical splice sites in both exons and introns, demonstrating that novel types of genetic variation affect splicing [43].

Though splicing MPRA have not yet been used to validate neurodevelopmental disorder-associated variant function, alternative splicing is a critical process for neuronal fate specification during neurogenesis [59, 60], and differences in alternative splicing have been identified in postmortem brains from individuals with autism, schizophrenia, and bipolar disorder [61]. Rare neurodevelopmental disorders can also be caused by alterations in alternative splicing. For example, familial dysautonomia, a degenerative sensory and autonomic nervous system disorder, is caused by a 5′ splice site mutation in an intron of *IKBKAP* [62]. The mutation results in variable exclusion of exon 20 and reduced IKAP protein levels in neuronal tissue [63]. Identifying the mutation has allowed understanding of the disease mechanism [64] and testing of therapeutic treatments [65, 66]. Therefore, splicing MPRA have great potential to identify variants that contribute to abnormal splicing in neurodevelopmental and neuropsychiatric disorders.

#### RNA editing

RNA sequences can be modified posttranscriptionally in a process known as RNA editing, which can alter the function of a gene [67, 68]. Dysregulation of RNA editing has been shown in many nervous system disorders such as brain cancer, addiction, depression, Alzheimer’s disease, amyotrophic lateral sclerosis (ALS), ASD, and intellectual disabilities (ID) [68, 69]. MPRA have been adapted to quantify RNA editing events such as uridine to pseudouridine (Ψ) which changes RNA regulation and stability [44, 70]. RNA editing MPRA consists of a promoter, coding sequence (CDS), putative Ψ sequence containing variants within the 3′ untranslated region (UTR), and a unique barcode (Fig. 3F) [44]. Once the MPRA library is introduced into cells, it is treated with a molecule that binds to the Ψ and prevents reverse transcription at the binding site [44]. Consequently, the exact base pair location of the Ψ alteration can be determined via high-throughput sequencing. This type of assay can show which underlying DNA sequences and variants lead to uridine to Ψ RNA editing, decoding much of the unknown regulatory code for RNA editing.

#### RNA stability and translation

In addition to transcriptional regulation, mRNA stability and translational control are another critical step that determines protein abundance. 5′ and 3′ UTRs are regulatory regions of the DNA that influence mRNA stability, localization, and translation [71, 72]. MPRA can be used to study the function of both 5′ and 3′ UTR sequences.

5′ UTRs affect translational efficiency by altering ribosome loading [73]. In an example of 5′ UTR MPRA, Sample et al. measured the impact of 5′ UTR sequences on ribosome loading by having putative 5′ UTR sequences inserted upstream to a GFP and a 3′ UTR [73]. After introducing the constructs to the cells, 5′ UTR sequences that are actively being translated in ribosomes are directly sequenced via polysome profiling [73]. This method identified 45 variants associated with disease that significantly affected ribosome loading [73]. 5′ UTR MPRA is an effective method to identify disease-associated variants that alter translational efficiency.

MPRA are also used to understand how variants within 3′ UTR sequences affect stability of mRNA (Fig. 3G) [74, 75]. In the 3′ UTR design, a promoter drives GFP expression which has a 3′ UTR containing a variant, and a unique barcode matched to that variant. Barcoded expression of GFP and 3′ UTR can be used to assess differences in RNA abundance (and hence stability). Lagunas et al. used this approach to assess the activity of > 500 de novo noncoding variants identified by WGS of ASD families, yielding 41 variants with differential stability effects in the brain [74]. This is in line with the previous findings that modifications in the 3′ UTR are broadly linked to brain function and neurodevelopmental disorders [76–78].

#### RNA localization

Location of RNA within the cell often closely aligns with its function. Massively parallel RNA assay (MPRNA) can test subcellular localization (e.g., nuclear vs. cytosolic) of long noncoding RNAs (lncRNAs) at scale [45]. The MPRNA construct consists of a cytosolic-localized *Sox2* variant (*fsSox2*), a DNA sequence that encodes a lncRNA, and a unique barcode (Fig. 3H) [45]. The *fsSox2* makes the baseline localization cytosolic; therefore, if the lncRNA creates a nuclear localization sequence, it will translocate to the nucleus. Once cells are transfected with the MPRNA construct, the cytoplasm and nucleus are isolated via subcellular fractionation [45]. The resulting barcode counts from RNA-seq inform lncRNA sequences that influence nuclear localization, which in turn affects the regulatory activity of the lncRNA [45]. Subcellular localization provides valuable insight into the function of lncRNAs, which is unknown compared to protein coding genes despite evidence found for their role in brain development and neurodevelopmental disorders such as ASD, Rett’s syndrome, attention-deficit hyperactivity disorder (ADHD), and schizophrenia [79–92] and other neurological disorders such as Alzheimer’s, Parkinson’s, and Huntington’s disease [82, 93].

### Limitations of MPRA

#### MPRA cannot identify target genes

Though MPRA is an incredibly useful tool to experimentally verify variant function, this assay is not without limitations. Enhancer, promoter, and silencer MPRA can effectively identify variants and elements with regulatory activity, but these assays cannot inform which gene(s) that the variants act on. Therefore, MPRA results need to be combined with other functional genomic data such as eQTLs and chromatin interaction profiles (Hi-C) to identify target genes [94]. While functional genomic approaches can be a good starting point to discern variant function, acquiring functional genomic datasets that match the appropriate biological context can be challenging when a rare cell type or environmental perturbation is used.

A complementary approach to address this gap is Perturb-seq, which employs a pooled library of gRNAs associated with a unique barcode that modulates expression of the gRNAs’ target genes. A Perturb-seq gRNA library could be designed for the MPRA-validated regulatory elements and introduced into a cell line expressing Cas9 protein. Introduced gRNAs can perturb the region of interest, and transcriptomic alterations can be profiled via scRNA-seq. The resulting data can explain the regulatory impact of perturbed elements within a given cell [95]. Moreover, because Cas9 perturbs host genomic DNA, it can further verify element function within the biologically relevant (epi)genetic context. Therefore, Perturb-seq can complement MPRA by shedding light on which genes and pathways MPRA-validated elements act on.

#### Genomic context

Another disadvantage of MPRA is that it uses exogenous DNA constructs that do not model the endogenous regulatory environment at the variant location. Exogenous DNA constructs are either episomal or inserted into the DNA at random locations. An episomal construct is not subject to cis-regulatory effects such as chromatin accessibility and conformation. Instead, its activity is only modulated by trans-regulatory effects, like transcription factors. In contrast to episomal MPRA, MPRA delivered through a lentiviral vector (LentiMPRA) randomly integrates into the host genome, hence enabling functional characterization of regulatory elements and variants within the context of the host genome [96]. However, LentiMPRA constructs are randomly integrated into the genome, so the (epi)genomic context of the integrated site most likely differs from that of the host genome and may differ from one insert to another.

The effect of genomic context has been evaluated with a new technique called PatchMPRA [97]. This technique leverages a cell line that has multiple known landing pads, each labeled with a unique genomic barcode. Because chromatin architecture of each landing pad has been well characterized, (epi)genomic context can be accounted for when interpreting the MPRA results. Maricque et al. used PatchMPRA to test over 30,000 combinations of CREs and local chromatin architecture in K562 cells [97]. They found that the location of landing pads in the genome had significant effects on barcode expression [97]. In particular, the DNA sequence of CREs determines the intrinsic regulatory activity, which is then fine-tuned by the chromatin environment [97]. While PatchMPRA enables systemic interrogation of interaction between regulatory elements and genomic contexts, it requires landing pads to be inserted into the host genome, which may not always be possible in some model systems.

### Future directions

#### Choosing cell type and developmental time period

**Fig. 4:**
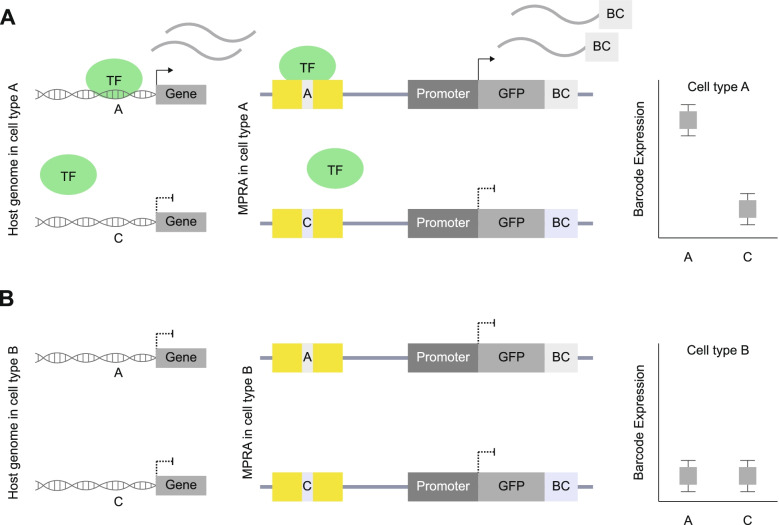
An example of how cell types influence variant function. **A** In cell type A, TF is expressed and binds to the A allele in both the host genome and the MPRA construct. In the host genome with allele A, the gene is expressed. In the MPRA construct with allele A, the barcoded (BC) reporter gene is expressed. TF does not bind to the C allele, so the gene and BC associated with that allele are not expressed. **B** In cell type B, TF is not expressed, so the allele is not associated with BC expression

Regulatory elements are often only functional within a given tissue or cell type [25, 98–101]. As regulatory elements display extensive tissue and cell type specificity [25, 98–101], it is important to choose the cell and/or tissue type carefully for an MPRA experiment. MPRA may give different results as to which CREs and variants have regulatory effects based on which cell type the MPRA is tested in (Fig. 4) [102]. This can be due to TFs being differentially expressed in different cell types [103] leading to cell type-specific regulatory element activity [25].

MPRA is most commonly performed, out of convenience, on cell types easily cultured and transfected in a lab (such as HEK293 cells [19]). Because TFs may only be expressed within specific cell types, MPRA results may change in other cell types (Fig. 4), so using a relevant brain cell type for neurodevelopmental disorders is a preferred experimental design. Genetic variation associated with multiple neurodevelopmental disorders is enriched in regulatory elements present in dorsal telencephalic neural progenitors and excitatory cortical neurons, making them optimal cell types to conduct MPRA experiments [25, 60, 98, 104–106] (Fig. 4).

The cell type specificity of MPRA has been previously demonstrated with an MPRA on random CREs tested in two cell lines: U87 glioblastoma cells and induced pluripotent stem cell (iPSC)-derived human neural progenitor cells (hNPCs) [102]. This study found a significant inverse correlation (*R* = −0.326) between regulatory activity of CRE barcode expression in these two cell types [102]. The difference in regulatory activity was attributed to the difference in TFs expressed in those cells and the presence of their binding motifs within the MPRA library. An enrichment in binding sites for TFs involved in brain development, such as SOX2, DBX1, and FOXP2, was found in hNPCs compared to U87 cells, as these TFs are more highly expressed in hNPCs [102]. These results underline the importance of choosing a relevant model system for studying brain-specific variant function.

To delineate variant effects on gene regulation in a cell type-specific manner in vivo, Cre recombinase-dependent MPRA packaged within an adeno-associated virus (AAV) has been developed [74]. In a preprint describing this method, mouse lines expressing Cre recombinase from an endogenous cell type-specific promoter is used to restrict the expression of an MPRA construct to a given cell type and location. This design has been used to test MPRA within excitatory neurons by injecting the Cre-dependent AAV MPRA into the cortex of Vglut1-IRES2-Cre-D mice [44]. Controlling for cell type specificity within an in vivo system is especially important as brain tissue is composed of heterogeneous cell types, and MPRA results from bulk tissue may mask the effects of variants functional in relatively less abundant cell types.

Just as cell and tissue types are important to MPRA, the development stage is a critical factor to consider when performing MPRA. This is especially important in studying genetic etiology of neurodevelopmental and psychiatric disorders which, by definition, have neurodevelopmental origin [60, 105, 107]. As such, investigating variant function during prenatal time periods when processes such as neurogenesis, gliogenesis, synaptogenesis, and pruning are occurring increases the likelihood of gaining neurodevelopmental relevant information via MPRA [108].

#### Response MPRA: gene-environment interactions explored

**Fig. 5:**
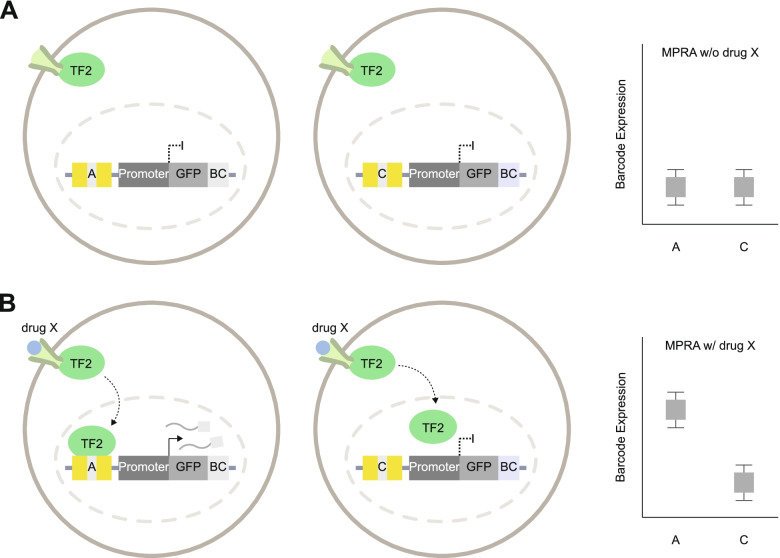
A cartoon example of a response MPRA. **A** In traditional MPRA, MPRA constructs introduced to the cells are not expressed because TF2 that acts on the element of interest is not translocated to the nucleus. Barcode expression of these alleles is displayed in a box plot. **B** In response MPRA, cells are treated with a drug X that activates downstream pathways to translocate response-dependent TF2 to the nucleus. Allele C disrupts the TF2 binding motif, and its unique barcode is not expressed, while allele A matches the TF2 motif leading to expression of its unique barcode. The barcode expression of each allele is displayed in an accompanied box plot. Therefore, this variant displays allelic regulatory activity only in response to drug X treatment

One critical, unanswered question in the field is the extent to which variant function is influenced by gene-environment interactions. External stimuli can alter a cellular pathway that has downstream effects on TF abundance and binding properties. Applying MPRA in this context can uncover a new class of variants that gain (or lose) regulatory effects upon exposure to external stimuli. Here, we propose a term “response MPRA” to describe MPRA performed in response to an external stimulus, such as exposure to hormones, drugs, or other biomolecules, as well as measuring gene regulatory effects in the context of a particular cell state (Fig. 5).

In an example of a response MPRA, Mulvey et al. [32] interrogated allelic regulatory activity of major depressive disorder (MDD) risk variants in response to all-*trans* retinoic acid (ATRA). ATRA, an acid derivative of retinol, activates the retinoic pathway, which has been implicated with the risk for MDD [32]. ATRA administration to N2A neuroblastoma cells not only increased the magnitude of allelic regulatory activity of a subset of variants with retinoic receptor motifs but also unmasked allele-specific activity not otherwise detected in the traditional MPRA [32]. As retinoic acid is a potent driver of neuronal differentiation, further investigation is required to distinguish ATRA-dependent regulatory effects from cell type-specific regulatory effects. Still, the evidence suggests that a subset of variants may function only upon activation of the retinoic pathway, providing a biological context for MDD genetic risk factors.

An additional application of the response MPRA follows the paradigm of measuring gene regulatory activity in the context of a particular biological process. In neurons, a classic example of this involves measuring CRE response upon stimulation with known modulators of neuronal activity. In an early adaptation of this approach, Nguyen et al. compared promoter and enhancer activities in neurons treated with potassium chloride with their unstimulated controls [109]. While similar sequences were found to be associated with increased neuronal activity between promoters and enhancers, the authors identified specific TF binding sites enriched in promoters that led to greater overall activity in response to neuronal activity. Another important cellular process relevant to neurodevelopment is the proliferation of neural progenitor cells. Dysregulation of the cell cycle and increased proliferation have been associated with brain overgrowth phenotypes present in individuals with ASD [110, 111]. By isolating proliferating cells, marked by incorporation of thymidine analogous such as BrdU, one can identify elements specifically active during phases of the cell cycle. These examples demonstrate how MPRA can be used to assess the importance of cell state in mediating gene regulatory activity.

Given the limited therapeutic options available for neurodevelopmental and psychiatric disorders, response MPRA can provide a high-throughput framework to investigate the impact of a drug candidate on variant function, potentially leading to high-throughput testing for pharmacogenomics and personalized therapies based on genetic background. An example of such an approach could be testing ADHD-associated variants with methylphenidate (a common ADHD medication [112]) to determine which variants have altered functionality upon drug exposure. Identifying the alleles that are responsive to methylphenidate exposure may eventually lead to more informed clinical decisions based on patient genotype during ADHD treatment. Choosing an appropriate model system with disease relevance is critical to accurately assess drug response. In this regard, patient-derived iPSCs are ideal tools for drug discovery efforts as iPSCs can differentiate into many cell types and can be scaled to meet coverage requirements for MPRA screening.

Finally, response MPRA can provide a useful tool to study gene-environment interactions by interrogating the variant function upon exposure to environmental factors associated with disease risk. Maternal exposure during pregnancy to valproic acid (VPA), a commonly prescribed antiepileptic medication, has been associated with risk for ASD [113], as well as several other neurodevelopmental disorders [114–116]. Studies have shown that VPA exposure can alter the proliferation and neurogenic capacities of neural progenitor cells during brain development [117], which can result in downstream deficits in brain structure [118] and cytoarchitecture [119]. Performing VPA-response MPRA within a progenitor cell type using ASD-associated variants could shed light on which alleles have altered function upon VPA exposure. However, VPA is only one of many environmental factors that have been associated with ASD risk [120]. Overall, various classes of external stimuli, the number of variants whose function is altered upon stimulation, and the magnitude of these effects on gene regulation have yet to be explored and can provide novel insights into disease mechanisms.

## Conclusions

Many variants in noncoding regions have, and will continue to be, identified by large-scale studies such as GWAS, WGS, and QTL. Understanding the function of those variants, and which variants within a haplotype block are causal, is the next key step in moving from association to biological understanding. In this review, we outlined how MPRA can validate variant function in a wide range of regulatory elements such as enhancers, promoters, silencers, and TF binding sites. We also described how MPRA can be used to garner mechanistic understanding of posttranscriptional regulation such as splicing, RNA modification, RNA stability, translation, and RNA localization. MPRA results can change based on cell type, stimulus state, and developmental time period, so these parameters must be carefully considered when designing an MPRA experiment. MPRA has limitations in associating regulatory effects to a target gene and lacks epigenetic context. Complementary approaches that range from other functional genomic resources (e.g., eQTLs, ASCA, and Hi-C) to other screening platforms (e.g., Perturb-seq) will extend knowledge gained from MPRA to provide a greater understanding of the mechanisms by which genetic variants affect brain structure, function, and development.

## Data Availability

Not applicable

## Abbreviations

AAV: Adeno-associated virus
ADHD: Attention-deficit hyperactivity disorder
AF: Allele frequency
ALS: Amyotrophic lateral sclerosis
ASCA: Allele-specific chromatin accessibility
ASD: Autism spectrum disorder
ATR: All-trans retinoic acid
BC: Barcode
CDS: Gene coding region
ChiP-Seq: Chromatin immunoprecipitation sequencing
CMC-N: Cyclohexyl-N′-(β-[N-methylmorpholino]ethyl)carbodiimide p-toluenesulfonate salt
CRE: Cis-regulatory element
CRISPR: Clustered regularly interspaced short palindromic repeats
dCas9: Catalytically dead Cas9 endonuclease
dCas9-KRAB: Dead Cas9 protein with an attached Krüppel-associated box
DDHD2: DDHD domain containing 2
DNA: Deoxyribonucleic acid
DNA-seq: Deoxyribonucleic acid sequencing
FACS: Fluorescence-activated cell sorting
GFP: Green fluorescent protein
GPRA: Gigantic parallel reporter assay
gRNA: Guide RNA
GWAS: Genome-wide association study
hNPCs: Human neural progenitor cells
ID: Intellectual disability
iPSCs: Induced pluripotent stem cells
LD: Linkage disequilibrium
lncRNA: Long noncoding RNA
MDD: Major depressive disorder
MPRA: Massively parallel reporter assay
MPRNA: Massively parallel RNA assay
mRNA: Messenger RNA
P2A: Peptide 2A
QTL: Quantitative trait loci
RNA: Ribonucleic acid
RNA-seq: RNA sequencing
RT: Reverse transcription
RT-PCR: Reverse transcription-polymerase chain reaction
SCP1: Super core promoter 1
SNP: Single-nucleotide polymorphism
STARR-seq: Self-transcribing active regulatory region sequencing
TF: Transcription factor
TFBS: Transcription factor binding site
TransMPRA: Transcription factor binding MPRA
UTR: Untranslated region
VPA: Valproic acid
WES: Whole exome sequencing
WGS: Whole genome sequencing
YFP: Yellow fluorescent protein
Ψ: Pseudouridine
